# Cancer: looking for simplicity and finding complexity

**DOI:** 10.1186/1475-2867-6-4

**Published:** 2006-02-15

**Authors:** Fabio Grizzi, Maurizio Chiriva-Internati

**Affiliations:** 1 Laboratori di Medicina Quantitativa, Istituto Clinico Humanitas IRCCS, 20089 Rozzano, Milan, Italy; 2 "Michele Rodriguez" Foundation, Scientific Institute for the Quantitative Measures in Medicine, 20054 Milan, Italy; 3 Department of Microbiology & Immunology, Texas Tech University Health Sciences Center and Southwest Cancer Treatment and Research Center, Lubbock, Texas 79430, USA

## Abstract

Cancer is one of the most complex dynamic human disease. Despite rapid advances in the fields of molecular and cell biology, it is still widely debated as to how neoplastic cells progress through carcinogenesis and acquire their metastatic ability. The need to find a new way of observing anatomical entities and their underlying processes, and measuring the changes they undergo, prompted us to investigate the *Theory of Complexity*, and to apply its principles to human cancer. Viewing a neoplasm as a system that is complex in *time *and *space *it is likely to reveal more about its behavioral characteristics, and this manner of thinking may help to clarify concepts, interpret experimental data, indicate specific experiments and categorize the rich body of knowledge on the basis of the similarities and/or shared behaviors of very different tumors.

## Introduction

Carcinogenesis has long been thought to be a *multi-step *process [1]; however, it has only recently become possible to identify a large number of the molecular events underlying the *initiation *and *progression *of different human tumors [2]. After a quarter century of rapid advances, cancer research has generated an intricate body of knowledge showing that cancer is a disease that involves dynamic changes in the genome [3].

The foundations of this knowledge were mainly laid by the discovery of genomic alterations or *mutations *that produce *oncogenes *with a dominant gain of function and *tumour-suppressor genes *with a recessive loss of function. Both of these cancer gene classes were identified on the basis of their alterations in human and animal neoplastic cells, and their elicitation of cancer phenotypes in experimental models [4-7].

Although considerable advances have been made in terms of our molecular and cellular knowledge, very little is understood about the *physics *underlying human carcinogenesis. It is now well known that the conception of *anatomical entities *as an infinite hierarchy of infinitely graduated forms and the increasing discoveries of functional variables have generated a growing awareness of *complexity*, thus highlighting new and exciting properties of organized biological matter [8].

More than *100 *distinct types of human cancer have been described, and subtypes of tumors can be found within specific organs. Cancer is increasingly recognized as being a highly *heterogeneous disease *within individual tumors, and within and between tumour types [9]. This heterogeneity is manifested at both genetic and phenotypic level, and primarily determines the *self-progression *of neoplastic disease and its response to therapy.

The discovery of this increasing complexity has led many researchers to ask a number of stimulating questions. How many distinct regulatory circuits within each type of target cell must be disrupted in order to make it become cancerous? Does the same set of cell regulatory circuits suffer disruption in the cells of the disparate neoplasms arising in humans? And, if we had a complete description of all of the molecular reactions occurring within a living normal cell and its tumoral counterpart, would we understand that cell?

**Figure 1:**
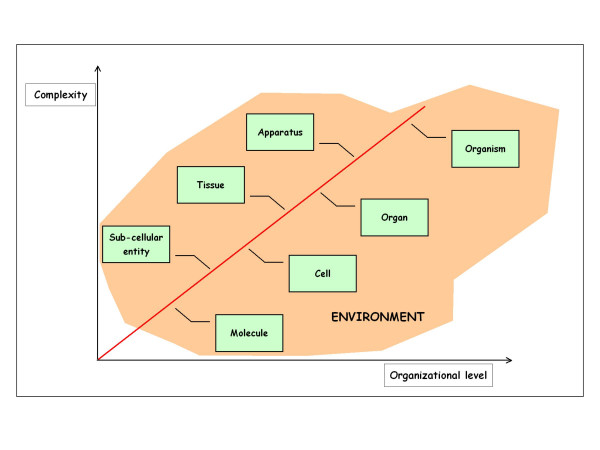
Human beings are complex hierarchical systems consisting of a number of hierarchical levels of anatomical organization (genes, sub-cellular entities, cells, tissues, organs, apparatuses, and organism) that interrelate differently with each other to form networks of growing complexity. Each anatomical entity is embedded in a macro-environmental system that influences the general behaviour of the entity itself.

Scientists seldom give much thought to such questions, and so it was an unusual gathering that assembled in 1997 in order to discuss how far a *reductionist approach *can take biology [10]. *Reductionism *seeks to explain the wide variety of natural phenomena on the basis of the behavior of a limited number of simpler constituents subject to rigorous and simple laws [8]. It has been a powerful driving force in science, and its success is plainly evident in the impressive triumphs of molecular biology that have allowed us to understand the molecular basis of such different areas as developmental and cell biology, immunology, and general and systemic human pathology [10,11]. However, the question remains as to how to transform this molecular knowledge into an understanding of the complex phenomena existing in *genes*, *sub-cellular entities*, *cells*, *tissues*, *organs*, *apparatuses *and *organism *(Figure 1) [12-22].

The need to tackle *system complexity *has become even more apparent since the completion of the various genome projects, an approach that has stimulated a search for new ways of developing our quantitative understanding of the complex processes underlying cancer initiation, progression and metastasis. The pioneering words of Sydney Brenner ("In the next 25 years, we are going to have to teach biologists another language....I don't know what its called yet; nobody knows....") can today be considered a manifestation of "modernity" [23,24].

A quantitative *system-level *understanding of cancer biology basically requires a mathematical framework that is capable of describing the principles governing the *structure *and *behavior *of a tumour [25,26]. Moreover modeling the growth and development of human tumors using mathematics and biological data has become a burgeoning area of cancer research [27-34]. Mathematical models represent a compulsory tool for organizing the large amounts of data concerning the genetic and biochemical pathways of cancer, and providing an advanced interpretation of its dynamics and control.

We here discuss cancer as a complex dynamic disease, and introduce some of the critical concepts necessary to give meaning of its underlying physical complexity.

## Cancer is a dynamic system

Carcinogenesis is one of the most complex phenomena in biology. Cancer is a *dynamic system *that is discontinuous in *space *and *time*, but advances through qualitatively different *states*: *i.e*. the configuration of a system at any particular instant that is specified by a great number of dynamic variables [35].

**Figure 2:**
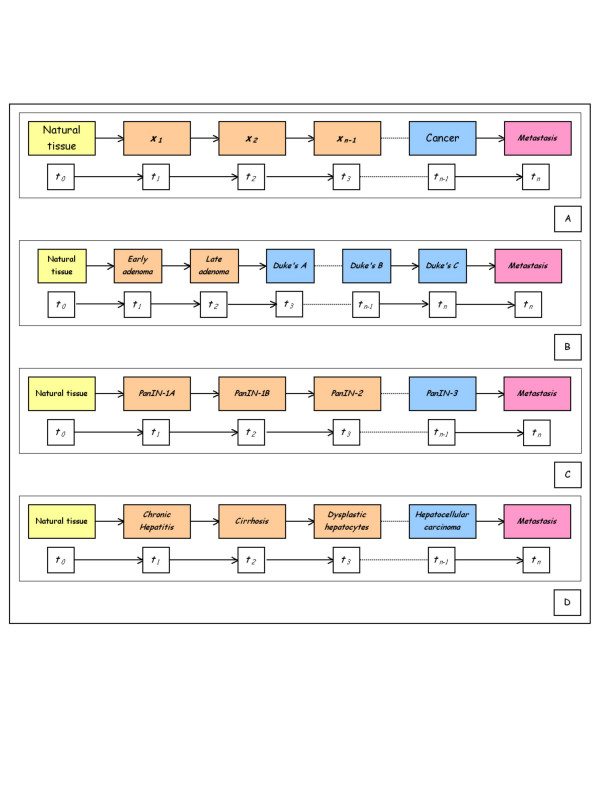
Multi-state carcinogenesis: *a*) the schema shows the progression of different qualitative states (*x*_1_, *x*_2_....*x*_*n*-1_, *x*_*n*_) identifiable in the development of cancer from normal tissue. The time parameter (*t*_0_, *t*_1_... ....*t*_*n*-1_, *t*_*n*_) depends on a large number of variables interconnected in many ways in a non-linear manner. This makes it extremely difficult to predict the exact time interval between two successive states. Although carcinogenesis is a *continuum*, its differentiation into successive states is still based on differences in histological and clinical data. Examples of dynamical view of colorectal (*b*) pancreatic (*c*) and liver (*d*) carcinogenesis [38,50,51].

In mathematical terms, a dynamic system depends on a set of different states or possible *configuration patterns *(*x*), and a number of *transitions *or *steps *() from one state to another during a certain interval of time (*t*). When the transitions are caused by a generating factor (*u*), the temporal behavior of the system can be theoretically described using general equation 1:

 = f(x, u, t)     1)

where *f *is a continuous non-linear function and the dot denotes a differentiation with respect to time (*t*) [35-37]. The parameter *time *depends on a large number of variables that are non-linearly interconnected in a multitude of ways, thus making it extremely difficult to predict the exact time interval between two successive states (Figure 2).

These states may or may not proceed to a more advanced state [35].

The continuous generation of *unstable states *during the course of carcinogenesis (initiation → progression → metastasis) has led to every sort of reorganization of different entities due to a change in the parameters on which they depend being physically defined as a *bifurcation*, whereas the term *catastrophe *describes an abrupt change that occurs as a sudden reaction of the system to a regular variation in external conditions [35-37]. In clinical terms, bifurcation could be used to describe a genomic mutation in a cell that drastically changes its behavior from normal to malignant, or a transformation from an *in situ *lesion to invasive cancer.

The genetic model for colorectal tumorigenesis originally proposed by Fearon and Vogelstein in 1990 was a pioneering view of cancer as a dynamic system [38]. Colorectal tumorigenesis proceeds through a series of genetic alterations that mainly involve oncogenes and tumour-suppressor genes. An additional defining feature of colorectal cancer is its *genetic instability *[29,39]. Two main types of genetic instability have been now identified: *a*) *microsatellite instability *leads to an increased point mutation rate, and *b*) *chromosomal instability *which refers to an enhanced rate of accumulating gross chromosomal aberrations [29,39].

Although the alterations usually occur at a characteristic state of tumour advancement, experimental evidence indicates that the ongoing accumulation of changes rather than their order of occurrence is more important in the course of cancer [38].

In physical terms, it is true that alterations in one parameter (*i.e*. chromosomal changes, DNA changes, specific gene changes or mitochondrial changes) are not necessarily associated with the loss of stability of a system, and it is also true that an *unstable system *is more sensitive to small changes in parameters (*i.e*. its state is more easily modifiable): in biological words, a growing network of cancer-susceptibility genes is formed as the neoplasm advances [35-41].

The human genome is typically so stable that the many genetic alterations required for cancer to develop cannot accumulate unless the rate of mutation increases to the point of making it genetically unstable [35,41].

Stelling *et al*. have used the term *robustness *to describe the ability of living systems to maintain performance (*phenotypic stability*) in the face of perturbations arising from environmental changes, stochastic events (or *intracellular noise*) and genetic variations [42], and cancer has been shown to be an extremely heterogeneous disease with a high level of robustness against a range of therapeutic effects [43-45].

## Cancer is a hierarchical system

The decisive step in carcinogenesis is the result of an irreversible qualitative change in one or more of the genetic characteristics of cancer cells. Although this modification governs the transformation of normal human cells into malignant cancer cells, it may or may not lead to visible changes in their *cytological *or *histological *structures [35]. This can be explained using the concept of *emergence*, which defines a human being as a complex system consisting of different anatomical entities that are interconnected at many organizational levels (*a hierarchical system*), have various degrees of complexity, and are governed by specific laws that only operate at a particular level (Figure 1).

Emergence is a seminal concept in *system theory*, where it denotes the principle that the "emergent" global properties defining *higher order systems *or "wholes" cannot generally be reduced to the properties of the *lower order subsystems *or "parts". We shall here use the word *emergence *to mean the appearance of *unexpected structures *and/or the occurrence of *surprising behaviors *in large systems consisting of microscopic (physical or biological) non-identical parts: *i.e*. structures and behaviors that are not intuitive or simply predictable [46-48].

In the experimental sciences, observed processes and patterns can often be conceptualized as *macro-scale *manifestations of *micro-scale *processes. However, in many cases, a more typical situation involves observed patterns or system states that are created or influenced by *multiple processes *and *controls *[49].

**Figure 3:**
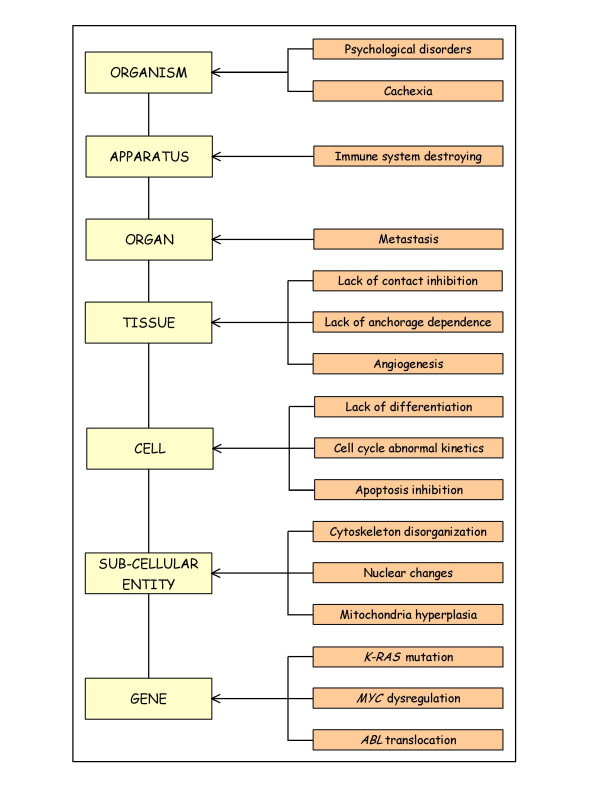
Hierarchical manifestation of human cancer. Neoplasia is a complex system determined by a number of processes acting at different molecular and cellular scales, by controls operating over much broader scales, and by factors such as structural controls that may operate at scales ranging from molecular to environmental. The schema exemplify some of the manifestations that can be found at different level of cancer complexity, all of them acting and governed by specific laws that only operate at a particular level.

Cancer is determined by a number of processes and controls operating over much broader scales, and by factors such as *structural controls *that may operate at scales ranging from molecular to environmental (Figure 3).

This *multiple scale causality *not only recognizes multiple processes and controls acting at multiple scales but, unlike a strict reductionist approach, may also recognize the fact that relevant "first principles" may reside at scales other than the smallest micro-scales. In other words, the observed phenomenon at each scale has structural and behavioral properties that do not exist at lower or higher organizational levels.

The results of several lines of research indicate that neoplastic cells share a common set of biological attributes (or "acquired capabilities") that operate and are controlled at different spatial and temporal scales, including their abilities:

*a*) to generate their own *mitogenic signals*;

*b*) to resist *exogenous growth-inhibitory signals*;

*c*) to evade *apoptosis*, and *senescence*;

*d*) to proliferate without limit;

*e*) to acquire vasculature (*i.e*. to undergo *angiogenesis*);

*f*) to *invade *and *metastasize *distant sites, *i.e*. organs (in more advanced cancers).

Aside from these shared behavioral characteristics and environmental conditions, it is necessary to emphasize that each cancer cell is a *self-governing entity*, which has the capability to progress independently by other surrounding cells.

## Cancer: looking for simplicity and finding complexity

Despite the rapid advances that have been made in the fields of molecular and cellular biology, there is no doubt that cancer is still now a very complex disease: it can be hypothesized that each tumour is unique, and that the spectrum of biological changes determining human tumors is infinitely variable.

As described above, carcinogenesis is a dynamic process that depends on a large number of variables and is regulated at multiple spatial and temporal scales, thus making it one of the most complex phenomena in biology. Carcinogenesis is a *non-linear *process, whose behaviour does not follow clearly predictable and repeatable pathways.

In *linear systems*, the relationship between an environmental factor increases, the system behaviour changes linearly in response to it. In contrast, behaviour of *chaotic systems i.e*. systems that exhibit variability, which may not be necessarily *random*, yet whose complex patterns are not discernible over a normal human time and spatial scale, may be perceived as unpredictable.

Periods of inactivity may be punctuated by sudden change, apparent patterns of behaviour may disappear and new patterns surprisingly emerge. Such behaviour emerges in complex systems, and are permanently sensitive to small perturbations. This chaotic behaviour does not indicate a lack of order. Rather, the order is difficult or impossible to describe in simple terms and requires complex narrative description.

However, non-linear systems are mainly characterised by three basic properties: (*a*) they do not react proportionally to the magnitude of their inputs; (*b*) they depend on their initial conditions. Small changes in the initial conditions may generate very different end points. (*c*) their behaviour is not deterministic.

According to non-linear mathematics, the carcinogenetic process can be defined as a system of evolving anatomical structures, each consisting of a virtually infinite number of interconnected parts and being governed by a large number of biological sub-processes that are different in time and space. In such a system: (*a*) the relationships between the *parts *are non-linear; (*b*) the variables influencing the behaviour of the system are interconnected in a complex manner; (*c*) the individual component parts show systematic heterogeneity; (*d*) small alterations in variables can lead to completely different outcomes; (*e*) the classical notions of *cause *and *effect *are replaced by concepts involving *control*, *bifurcation*, *energy *and *turbulence*.

The above characteristics are frequently shown by the fact that it is common to see differences in the progression or therapeutic response of the same tumour type, and the fact that cancer morphology does not always reveal an underlying biology.

Cancer does not conform to simple mathematical principles: the irregular mode of carcinogenesis, erratic tumour growth, variable response to tumoricidal agents, and poorly understood metastatic patterns constitute highly variable clinical behaviors.

In conclusion, the above reflections have led us to think that:

*a*) Cancer is a highly complex disease in time and in space.

*b*) In order to understand a problem that involves so many interacting systems within the same organism, we need to determine the type of data that needs to be collected at each level of organization, the boundary conditions to use when describing the disease (*i.e*. a perturbed system), and the technologies and approaches best suited to reveal its etiology.

*c*) Considering cancer as a robust system would provide us with a framework for future research strategies, and future cancer therapies may be judged on their ability to help control the robustness of tumors.

*d*) Modelling the growth and development of human tumours using mathematics and biological data is a burgeoning area of cancer research. Mathematical methods and their derivatives have proved to be possible and practical in oncology [31], but the current models are often simplifications that ignore vast amounts of knowledge: for example, most metabolic models seem to regard a cell as a bag of enzymes, and neglect its spatial heterogeneity and compartmentalisation [18]; furthermore, most models struggle to resolve the *10–12 *order-of-magnitude span of the timescales of systemic events, be they molecular (*ion channel gating*: 10^-6 ^sec), cellular (*mitosis*: 10^2^–10^3 ^sec) or physiological (*cancer progression*: 10^8 ^sec) [8].

Viewing cancer as a system that is dynamically complex in time and space will probably reveal more about its underlying behavioural characteristics. This way of thinking may further help to clarify concepts, interpret new and old experimental data, indicate alternative experiments and categorise the acquired knowledge on the basis of the similitude and/or shared behaviours of very different tumours. It is encouraging that mathematics, theoretics, biology and medicine continue to contribute together towards a common quantitative understanding of cancer complexity.
